# Real-world six-month outcomes after switching from aflibercept 2 mg to aflibercept 8 mg for neovascular age-related macular degeneration

**DOI:** 10.1007/s10384-026-01329-0

**Published:** 2026-02-03

**Authors:** Hiroya Kindo, Mio Morizane Hosokawa, Chihiro Ouchi, Ryo Matoba, Tetsuro Morita, Junko Hayashi, Yuki Morizane

**Affiliations:** Department of Ophthalmology, Graduate School of Medicine, Dentistry and Pharmaceutical Sciences, Okayama University, 2-5-1 Shikata-cho Kita-ku, Okayama City, Okayama 700-8558 Japan

**Keywords:** Aflibercept 8 mg, Neovascular age-related macular degeneration, Treat-and-extend, Switching, Treatment interval

## Abstract

**Purpose:**

To investigate 6-month outcomes in eyes with neovascular age-related macular degeneration (nAMD) switched from intravitreal aflibercept 2 mg to intravitreal aflibercept 8 mg.

**Study design:**

Retrospective observational study.

**Methods:**

We reviewed records of consecutive nAMD eyes switched from aflibercept 2 mg to 8 mg. In eyes continuing aflibercept 8 mg, best-corrected visual acuity (BCVA), treatment intervals, and anatomical/exudative parameters were evaluated at 6 months. In eyes that could not continue, reasons for discontinuation were examined.

**Results:**

Forty-four eyes from 44 patients were included. At 6 months, 35 eyes (79.5%) continued and 9 (20.5%) discontinued aflibercept 8 mg. Discontinuing eyes had significantly shorter pre-switch treatment intervals and more frequent prior therapies than continuing eyes. In the continuation group, BCVA remained stable (median 0.05 to 0.00 logMAR, *P* = 0.351), while the treatment interval was significantly extended (median 7.0 to 9.0 weeks, *P* < 0.001). Central retinal thickness and pigment epithelial detachment height decreased significantly (*P* = 0.035 and *P* = 0.021, respectively). The proportion of eyes with subretinal fluid significantly decreased from 74.3 to 37.1% (*P* = 0.003). Of the discontinuations, 4 were due to worsening exudation and 5 to inability to extend to ≥8 weeks as required by labeling. No intraocular inflammation or serious adverse events occurred.

**Conclusions:**

Switching to aflibercept 8 mg achieved anatomical improvements and longer treatment intervals in ~80% of nAMD cases, suggesting it may be a useful alternative to aflibercept 2 mg. However, continuation may be difficult in refractory cases requiring frequent injections before switching.

## Introduction

Age-related macular degeneration (AMD) is one of the leading causes of visual impairment in older adults worldwide, and untreated cases—particularly of neovascular AMD (nAMD)—can result in rapid vision loss [1]. Intravitreal anti-vascular endothelial growth factor (anti-VEGF) injections have become the standard treatment for nAMD and has greatly contributed to improving visual prognosis [1–4]. However, maintaining therapeutic efficacy requires frequent injections and hospital visits, which place a considerable burden on both patients and physicians [5, 6]. Therefore, establishing treatment regimens that can maintain therapeutic efficacy while reducing treatment frequency is a critical issue in the management of nAMD.

As one approach to address this issue, the treat-and-extend (TAE) regimen—in which treatment intervals are adjusted based on disease activity—has recently been adopted. While TAE enables extension of treatment intervals in some patients, others still require frequent injections at short intervals, and in such cases the reduction in treatment burden is not fully achieved [7–9]. For such patients, switching from one anti-VEGF agent to another is considered one treatment option, and in recent years, new anti-VEGF agents such as brolucizumab, faricimab, and aflibercept 8 mg, also referred to as high-dose aflibercept, have become available and may be potential options for switching.

Aflibercept 8 mg is an intravitreal formulation that delivers a fourfold higher molar dose into the vitreous compared with the conventional aflibercept 2 mg formulation; it was approved in the United States in 2023 and in Japan in 2024 following the CANDELA and PULSAR trials [10, 11]. In the PULSAR trial, aflibercept 8 mg demonstrated comparable visual gains to aflibercept 2 mg in treatment-naïve nAMD, and it was suggested that treatment intervals could be extended [11]. However, this evaluation was based only on treatment-naïve patients in a clinical trial setting, and in real-world practice, evidence regarding the effectiveness of switching to aflibercept 8 mg from other anti-VEGF agents remains limited. Switching patients from aflibercept 2 mg to aflibercept 8 mg is reported to show short-term efficacy [12, 13], but the overall effectiveness of this switch has not been fully evaluated.

In the present study, we evaluated the 6-month outcomes of switching from aflibercept 2 mg to aflibercept 8 mg in patients with nAMD in whom sufficient extension of treatment intervals could not be achieved with aflibercept 2 mg. To evaluate the effectiveness of this approach, patients were classified as either those who continued aflibercept 8 mg after the switch, whose 6-month outcomes were analyzed, and those who discontinued, for whom clinical courses were investigated.

## Materials and methods

This retrospective observational study was approved by the Institutional Review Board of Okayama University and adhered to the Declaration of Helsinki. Written informed consent was waived due to the retrospective design, and opt-out consent was implemented.

We retrospectively reviewed the medical records of consecutive patients with nAMD who were undergoing a TAE regimen and were switched from intravitreal aflibercept 2 mg to intravitreal aflibercept 8 mg between June and December 2024. Eyes were included if the treatment interval could not be extended beyond 10 weeks because of retinal exudation. Eyes were excluded if they underwent any ocular surgery potentially affecting visual acuity (e.g., cataract surgery or YAG capsulotomy) during the 6-month follow-up period.

After switching to aflibercept 8 mg, treatment was initiated at the same interval as that used at the time of the final aflibercept 2 mg injection, or at an interval shortened by 1–2 weeks. Thereafter, a TAE regimen was applied, in which the treatment interval was extended or shortened by 1–2 weeks depending on disease activity, without a loading phase at 4-week intervals. Treatment with aflibercept 8 mg was continued even if retinal exudation persisted, provided that exudation did not worsen relative to that during aflibercept 2 mg administration. According to the Japanese drug label requirements, aflibercept 8 mg must be administered at intervals of ≥8 weeks after the third injection. Therefore, if the treatment interval could not be extended to ≥8 weeks after the third aflibercept 8 mg injection, treatment with aflibercept 8 mg was discontinued and switched to another agent, including aflibercept 2 mg. Eyes that showed worsening of retinal exudation after switching to aflibercept 8 mg were also switched to alternative agents.

All of the patients had undergone a comprehensive ophthalmologic examination at each visit, including measurement of best-corrected visual acuity (BCVA) using a Landolt C chart, indirect ophthalmoscopy, slit-lamp biomicroscopy with contact lenses, and optical coherence tomography (OCT; swept-source OCT, DRI OCT-1 Atlantis, Topcon Corporation; or spectral-domain OCT, Spectralis, Heidelberg Engineering). When necessary, fundus photography (TRC-50DX; Topcon) was also conducted. The presence or absence of subretinal fluid (SRF), intraretinal fluid (IRF), pigment epithelial detachment (PED), and subretinal hemorrhage (SRH) was assessed using OCT images and fundus photographs. PED was defined to include all types, including fibrovascular, serous, and drusenoid PEDs. Central retinal thickness (CRT) was measured as the distance from the internal limiting membrane to Bruch’s membrane at the fovea. Central choroidal thickness (CCT) was measured as the distance from Bruch’s membrane to the choroid–sclera interface at the fovea. PED height, if present, was measured from the retinal pigment epithelium to Bruch’s membrane at the point of greatest elevation.

We investigated whether treatment with aflibercept 8 mg was continued at 6 months after the switch and compared characteristics between eyes that continued and those that discontinued therapy. For eyes that continued treatment, the date of the first aflibercept 8 mg injection was defined as baseline, and outcomes at 6 months were evaluated. Six-month outcomes were defined as the results obtained at the first aflibercept 8 mg administration performed after at least 6 months had passed since baseline. Changes from baseline to 6 months were assessed for BCVA, treatment interval, CRT, PED height, and CCT. Changes in PED height were analyzed only in eyes with PED at baseline. In addition, changes in the proportions of eyes with SRF, IRF, PED, and SRH were examined. For eyes that could not continue aflibercept 8 mg, the reasons for discontinuation and subsequent clinical course were reviewed.

BCVA was converted to logarithm of the minimum angle of resolution (logMAR) units for analysis. For comparisons of continuous variables between two independent groups, the Mann–Whitney U-test was used, whereas paired continuous variables were compared using Wilcoxon’s signed-rank test. Categorical variables between independent groups were compared using the chi-square test or Fisher’s exact test, as appropriate. Paired categorical variables were analyzed using McNemar’s test. All statistical analyses were performed using SPSS version 29 (IBM). A *P*-value of <0.05 was considered statistically significant, and results are presented as the median (interquartile range).

## Results

A total of 44 eyes from 44 patients were included in this study. No patients underwent bilateral switching to aflibercept 8 mg. The median age of patients was 76.5 (71.5–82.5) years, and 65.9% were men. Although 45 eyes met the inclusion criteria, one eye was excluded because cataract surgery was performed within 6 months of switching to aflibercept 8 mg. At 6 months, 35 eyes (79.5%) continued treatment with aflibercept 8 mg, while 9 eyes (20.5%) discontinued treatment. Eyes that discontinued aflibercept 8 mg had significantly shorter treatment intervals before switching compared to those that continued, and more frequently had a history of prior treatment with other anti-VEGF agents or photodynamic therapy (PDT) before aflibercept 2 mg. Among those with such prior treatment history, some could not achieve disease control with brolucizumab and/or faricimab at intervals of ≥8 weeks and therefore required aflibercept 2 mg every 4–6 weeks; this subgroup was also significantly more frequent in eyes that discontinued aflibercept 8 mg (4 eyes in the continuation group vs 5 eyes in the discontinuation group, *P* = 0.010). Table 1 provides a comparison of the baseline characteristics between eyes that continued and those that discontinued aflibercept 8 mg.

**Table 1:** Baseline clinical characteristics of patients who continued or discontinued aflibercept 8 mg treatment

	Continued	Discontinued	*P-*value
Eyes, n (%)	35 (79.5)	9 (20.5)	
Age, years (IQR)	77.0 (72.0–81.0)	79.0 (69.5–81.5)	0.886
Sex: male, n (%)	23 (65.7)	6 (66.7)	0.641
LogMAR BCVA (IQR)	0.05 (− 0.08–0.22)	0.15 (0.02–0.37)	0.111
CRT, μm (IQR)	287.0 (213.0–373.0)	393.0 (227.0–487.0)	0.125
CCT, μm (IQR)	213.0 (140.0–291.0)	182.5 (114.8–329.8)	0.787
PED height, μm (IQR)*	242.5 (171.8–329.5)	311.5 (220.3–515.0)	0.218
Subtypes of MNV: type1/type2/type3/PCV, n	18/2/0/15	3/0/0/6	0.395
Number of anti-VEGF injections before switching, n (IQR)	28.0 (7.0–50.0)	14.0 (7.0–64.5)	0.841
Treatment intervals before switching, weeks (IQR)	7.0 (6.0–9.0)	4.0 (4.0–5.0)	<0.001
History of prior treatment with other anti-VEGF agents or PDT before aflibercept 2 mg, n (%)	9 (25.7)	6 (66.7)	0.030

*IQR*, interquartile range; *LogMAR*, logarithm of minimum angle of resolution; *BCVA*, best-corrected visual acuity; *CRT*, central retinal thickness; *CCT*, central choroidal thickness; *PED*, pigment epithelial detachment; *MNV*, macular neovascularization; *PCV*, polypoidal choroidal vasculopathy; *VEGF*, vascular endothelial growth factor; *PDT*, photodynamic therapy

*PED height was evaluated only in eyes with PED (20 in the continuation group and 8 in the discontinuation group)

### Six-month outcomes in eyes that continued aflibercept 8 mg treatment

**Fig. 1:**
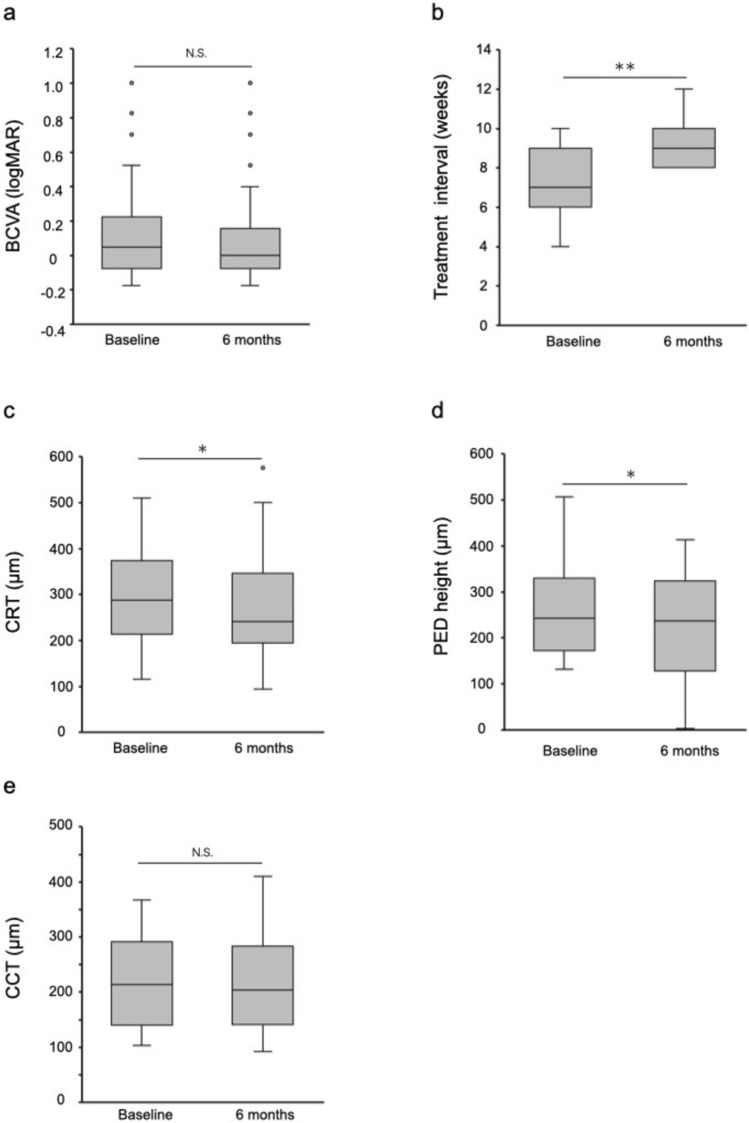
Six-month outcomes in eyes that continued aflibercept 8 mg. **a** Best-corrected visual acuity (BCVA); **b** Treatment interval; **c** Central retinal thickness (CRT); **d** Pigment epithelial detachment (PED) height; **e** Central choroidal thickness (CCT). ** *P* < 0.001, * *P* < 0.05, N.S. = not significant.

**Fig. 2:**
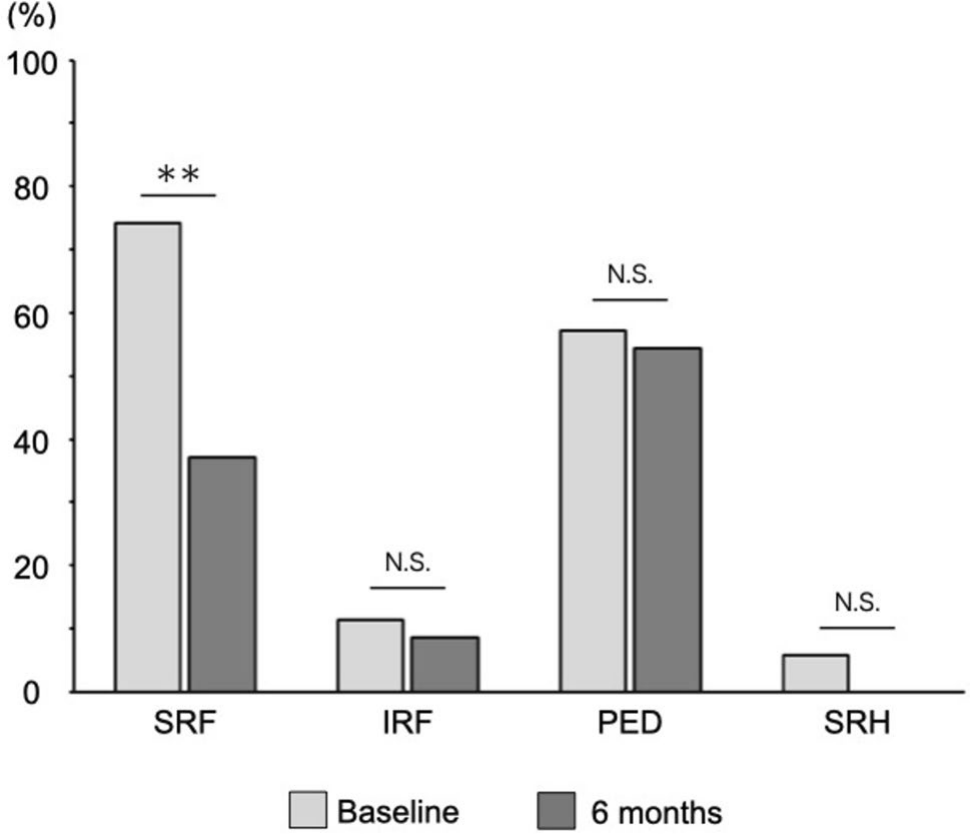
Responses of eyes treated with aflibercept 8 mg. Percentages of eyes with subretinal fluid (SRF), intraretinal fluid (IRF), pigment epithelial detachment (PED), and subretinal hemorrhage (SRH) are shown at baseline and at 6 months ** *P* < 0.001; N.S. = not significant.

**Fig. 3:**
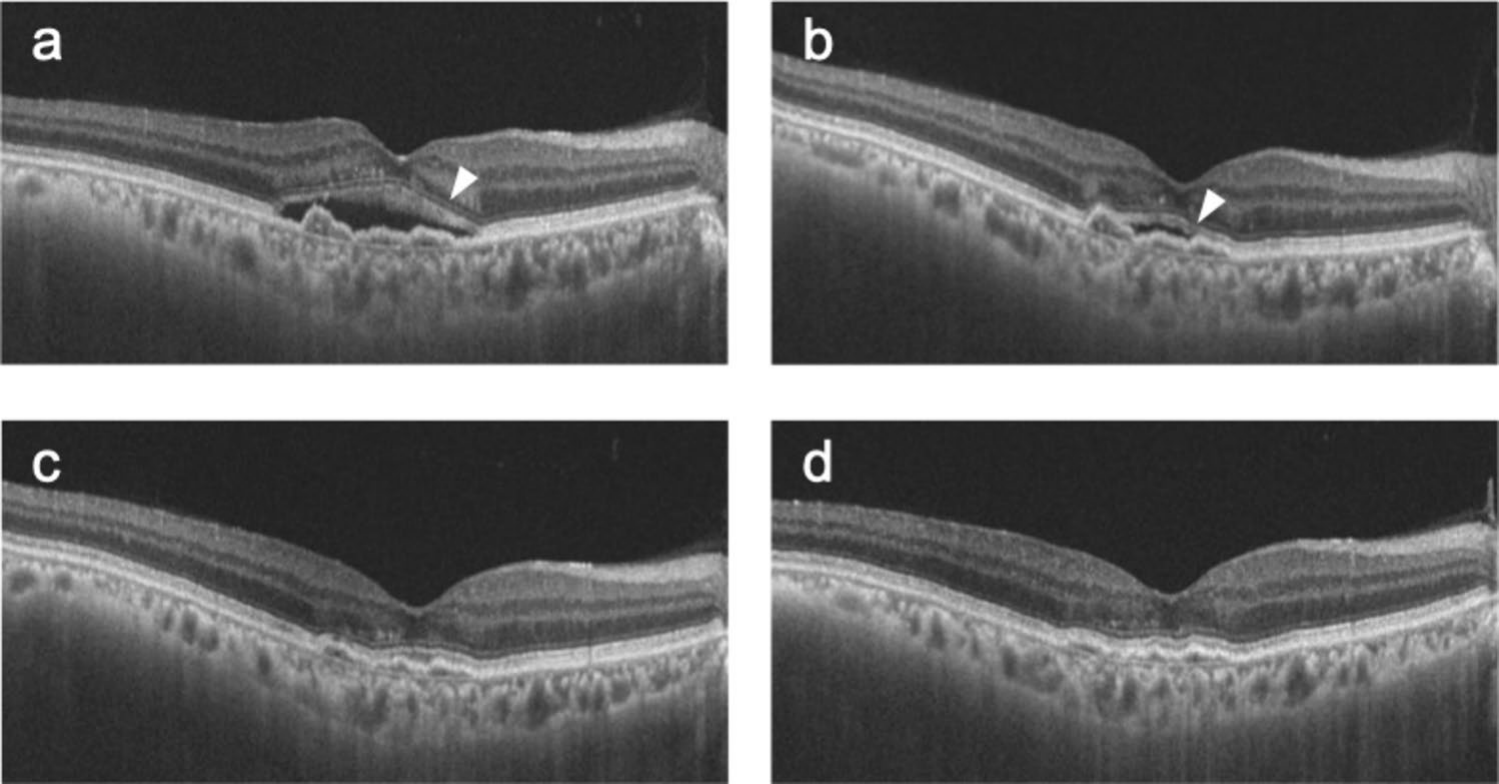
A representative case of a 71-year-old man with type 1 macular neovascularization in the right eye who continued treatment with aflibercept 8 mg for 6 months after switching from aflibercept 2 mg. **a** Nine weeks after the seventh injection of aflibercept 2 mg (baseline), subretinal fluid (SRF) was observed (arrowhead). Visual acuity was 0.7, followed by the first injection of aflibercept 8 mg. **b** Eight weeks after the first injection of aflibercept 8 mg, slight residual SRF was still present but had decreased (arrowhead). Visual acuity was 0.7, and the second injection of aflibercept 8 mg was administered. **c** Nine weeks after the second injection of aflibercept 8 mg, SRF had resolved. Visual acuity was 0.7, and the third injection of aflibercept 8 mg was administered. **d** Eleven weeks after the third injection of aflibercept 8 mg, no recurrence of SRF was observed. Visual acuity was 0.7, and the fourth injection of aflibercept 8 mg was administered.

In the 35 eyes that continued aflibercept 8 mg, BCVA remained statistically unchanged from 0.05 (−0.08 to 0.22) at baseline to 0.00 (−0.08 to 0.15) at 6 months (*P* = 0.351; Fig. 1a). The treatment interval was significantly extended from 7.0 (6.0–9.0) weeks at baseline to 9.0 (8.0–10.0) weeks at 6 months (*P* < 0.001; Fig. 1b). No eyes showed a shorter treatment interval at 6 months than the interval prior to switching. CRT significantly decreased from 287.0 (213.0–373.0) μm at baseline to 240.0 (194.0–346.0) μm at 6 months (*P* = 0.035; Fig. 1c). PED height was analyzed in the 20 eyes with PED at baseline and significantly decreased from 242.5 (171.8–329.5) μm at baseline to 226.5 (127.8–323.0) μm at 6 months (*P* = 0.021; Fig. 1d). No eyes showed new PED at 6 months. There was no statistically significant change in CCT from 213.0 (140.0–291.0) μm at baseline to 203.0 (141.0–283.0) μm at 6 months (*P* = 0.207; Fig. 1e). The proportions of eyes with SRF, IRF, PED, and SRH were 74.3%, 11.4%, 57.1%, and 5.7% at baseline, and 37.1%, 8.6%, 54.3%, and 0.0% at 6 months, respectively. Among these, only the proportion of eyes with SRF significantly decreased (*P* = 0.003; Fig. 2). A representative case is shown in Figure 3.

### Outcomes in eyes that discontinued aflibercept 8 mg treatment

The reasons for discontinuation of aflibercept 8 mg were: 4 eyes showed worsening of retinal exudation after switching to aflibercept 8 mg, and 5 eyes initially exhibited a reduction in exudation after switching but could not extend the treatment interval to ≥8 weeks after three injections of aflibercept 8 mg. All 4 eyes with worsening of retinal exudation had required treatment with aflibercept 2 mg at 4-week intervals before switching, and the worsening was observed after the first injection of aflibercept 8 mg. After discontinuation of aflibercept 8 mg, one eye was switched to faricimab, and the remaining 8 eyes were switched back to aflibercept 2 mg. Among the 4 eyes with worsening retinal exudation, one eye developed new SRH, one showed worsening of pre-existing SRH, one exhibited increased SRF, and one showed worsening of PED. In the eye in which SRH newly developed, subsequent vitreous hemorrhage occurred, necessitating vitrectomy. The eye with new SRH showed worsening of BCVA from 0.15 at baseline to 1.40 at 6 months, and the eye with enlargement of SRH also showed worsening of BCVA from 0.22 at baseline to 1.40 at 6 months. In the 2 eyes with worsening SRF or PED, and in the 5 eyes in which the treatment interval could not be extended to ≥8 weeks, BCVA did not decrease at 6 months.

### Adverse events

During the 6-month follow-up period, no intraocular inflammation (IOI), such as iritis or retinal vasculitis, was observed in patients treated with aflibercept 8 mg, and no serious adverse events, such as endophthalmitis, myocardial infarction, or stroke, were reported.

## Discussion

In the present study, at 6 months after switching from aflibercept 2 mg to aflibercept 8 mg, approximately 80% of patients were able to continue aflibercept 8 mg, with anatomical improvement and extension of treatment intervals observed in this group. In the ALTAIR study, which evaluated the 2-year outcomes of aflibercept 2 mg using a treat-and-extend regimen in Japanese patients with nAMD, more than 40% of eyes achieved the maximum treatment interval of 16 weeks, whereas 30–40% of eyes required the minimum treatment interval of 8 weeks, indicating that a certain proportion of cases have difficulty extending treatment intervals [8]. Our findings suggest that aflibercept 8 mg may offer a useful alternative for such cases. On the other hand, about 20% of eyes could not continue aflibercept 8 mg. Compared with those that continued treatment, eyes that discontinued had significantly shorter pre-switch treatment intervals and a higher frequency of prior treatment with other anti-VEGF agents or PDT before receiving aflibercept 2 mg. These results suggest that in more treatment-refractory nAMD cases—characterized by frequent injections and multiple prior therapies—long-term continuation of aflibercept 8 mg may be challenging. Taken together, these findings provide practical information for selecting patients likely to benefit from switching to aflibercept 8 mg.

Among the eyes that discontinued aflibercept 8 mg, 5 cases were unable to continue due to the requirements of drug labeling. In Japan, restrictions in the drug label and health insurance system mandate that after the initial three loading injections, aflibercept 8 mg must be administered at intervals of at least 8 weeks. Although details vary by country, similar system-based restrictions are reported outside of Japan as factors limiting continuation of aflibercept 8 mg [13]. In addition, 4 eyes that discontinued treatment showed worsening of exudative lesions, and in 2 of these, the onset or enlargement of SRH led to visual deterioration. A previous report describes a case in which SRH developed and subsequent visual decline occurred after switching from aflibercept 2 mg to faricimab [14]. These results suggest that there is a risk of exudative worsening when switching anti-VEGF agents. However, in this study, all 4 eyes with worsening had required aflibercept 2 mg every 4 weeks prior to switching and were highly active nAMD cases, suggesting that the risk of exudative worsening may have been present regardless of whether treatment was switched. Therefore, whether exudative worsening reflects the intrinsic severity of these cases or whether switching anti-VEGF agents itself poses a risk of disease progression requires further investigation in future prospective studies.

With respect to safety, no apparent IOI was observed during the 6 months after switching to aflibercept 8 mg in the present study. In the PULSAR trial, no clear difference in the incidence of IOI was demonstrated between the aflibercept 2 mg and aflibercept 8 mg groups; in the HARBOR trial, which investigated a higher dose of ranibizumab, no dose-dependent differences in adverse events are reported [11, 15]. On the other hand, real-world studies report that the incidence of IOI with aflibercept 8 mg may be higher than historically observed with aflibercept 2 mg [16, 17]. The precise mechanisms of IOI remain unclear, and careful monitoring and further research are warranted to clarify the impact of high-dose formulations on the occurrence of IOI.

The present study demonstrates trends similar to those previously reported in nAMD after switching anti-VEGF therapy from aflibercept 2 mg to brolucizumab or faricimab, including anatomical improvement, extension of treatment intervals, and maintenance of visual acuity [14, 18–20]. However, differences in patient background, treatment regimens, and outcome measures across studies make direct comparisons of post-switch outcomes among agents difficult. Brolucizumab is characterized by its small molecular size, which enables high molar dosing, and by its strong binding affinity to VEGF, although a relatively higher risk of IOI is reported [21, 22]. Faricimab, in contrast, is a bispecific antibody that simultaneously inhibits VEGF-A and angiopoietin-2 [23, 24]. Given these distinct pharmacologic profiles, future prospective trials, including head-to-head comparisons, will be required to optimize switching strategies among anti-VEGF agents. Meanwhile, this study reveals that cases previously unable to continue brolucizumab and/or faricimab were significantly more common in eyes that discontinued aflibercept 8 mg than in those that continued aflibercept 8 mg. This finding suggests that, in highly active nAMD, a subset of eyes may have difficulty achieving adequate disease control regardless of which anti-VEGF agent is used.

This study has several limitations. The most important is its retrospective design, due to which the timing of switching to aflibercept 8 mg was determined by each treating physician with patient consent. This reflects real-world practice but lacks standardized switching criteria. In addition, although multivariable analysis would be required to accurately identify factors associated with continuation of aflibercept 8 mg, the limited sample size precluded such an analysis in this study.

In conclusion, in patients with nAMD who could not achieve sufficient extension of treatment intervals with aflibercept 2 mg, switching to aflibercept 8 mg resulted in anatomical improvement and extended treatment intervals in approximately 80% of cases at 6 months, suggesting that aflibercept 8 mg may offer a useful alternative to aflibercept 2 mg. However, in patients with more treatment-refractory disease, characterized by frequent injections and multiple prior therapies before switching, continuation after switching may be challenging.
